# Image processing tools in the study of environmental contamination by microplastics: reliability and perspectives

**DOI:** 10.1007/s11356-022-22128-3

**Published:** 2022-07-28

**Authors:** Tommaso Valente, Daniele Ventura, Marco Matiddi, Alice Sbrana, Cecilia Silvestri, Raffaella Piermarini, Carlo Jacomini, Maria Letizia Costantini

**Affiliations:** 1 Department of Environmental Biology, La Sapienza’ University of Rome, P.le Aldo Moro 5, 00185 Rome, RM Italy; 2 ISPRA, Italian National Institute for Environmental Protection and Research, Via di Castel Romano 100, 00128 Rome, RM Italy; 3 PhD Program in Evolutionary Biology and Ecology, Department of Biology, University of Rome ‘Tor Vergata’, Via della Ricerca Scientifica snc, 00133 Rome, RM Italy

**Keywords:** Image analysis, Shape descriptors, Shape variations, Microplastic extraction, Microplastic characterization, Microplastic classification, Corrosiveness test

## Abstract

Microplastic pollution is one of the greatest environmental concerns for contemporary times and the future. In the last years, the number of publications about microplastic contamination has increased rapidly and the list is daily updated. However, the lack of standard analytical approaches might generate data inconsistencies, reducing the comparability among different studies. The present study investigates the potential of two image processing tools (namely the shapeR package for R and ImageJ 1.52v) in providing an accurate characterization of the shape of microplastics using a restricted set of shape descriptors. To ascertain that the selected tools can measure small shape differences, we perform an experiment to verify the detection of pre-post variations in the shape of different microplastic types (i.e., nylon [NY], polyethylene [PE], polyethylene terephthalate [PET], polypropylene [PP], polystyrene [PS], and polyvinylchloride [PVC]) treated with mildly corrosive chemicals (i.e., 10% KOH at 60 °C, 30% H_2_O_2_ at 50 °C, and 15% H_2_O_2_ + 5% HNO_3_ at 40 °C; incubation time ≈ 12 h). Analysis of surface area variations returns results about the vulnerability of plastic polymers to digestive solutions that are aligned with most of the acquired knowledge. The largest decrease in surface area occurs for KOH-treated PET particles, while NY results in the most susceptible polymer to the 30% H_2_O_2_ treatment, followed by PVC and PS. PE and PP are the most resistant polymers to all the used treatments. The adopted methods to characterize microplastics seem reliable tools for detecting small differences in the shape and size of these particles. Then, the analytic perspectives that can be developed using such widely accessible and low-cost equipment are discussed.

**Supplementary Information:**

The online version contains supplementary material available at 10.1007/s11356-022-22128-3.

## Introduction

In the twentieth century, *Homo sapiens* became able of producing a new class of totally synthetic materials, which he called plastics. All plastic materials are organic polymers that share low production costs, malleability, and durability (Geyer et al. 2017). Since the 1950s, plastic waste is accumulating in natural environments and today contaminates almost all places on earth (Zalasiewicz et al. 2016). Due to their insolubility, biochemical inertness, and high molecular weight, most of the plastic polymers show low toxicity (Worm et al. 2017). However, many plastic-associated chemicals — such as monomer residues, plasticizers, and pigments — are known to be hazardous, often toxic, or carcinogenic (Deanin 1975; Lithner et al. 2011; Fries et al. 2013). The lack of natural analogues makes plastic polymers resistant to biodegradation (Gómez and Michel 2013). Plastic waste may persist and accumulate for centuries both in marine and terrestrial environments (Kubowicz and Booth 2017). In the meantime, both environmental and biological drivers can split plastic litter into plastic particles of ever-smaller sizes, which may sorb many kinds of persistent pollutants due to their high surface-area-to-volume ratio (Wright and Kelly, 2017; Huang et al. 2021). Therefore, plastic pollution is recognized as one of the greatest environmental concerns of contemporary times and an emerging threat for the future (Horton et al. 2017).

Thompson et al. (2004) first coined the term “microplastic” (MP), which is now generally used to describe plastic fragments less than 5 mm in size (Arthur et al. 2009; Frias and Nash 2019). In recent years, several studies began to focus on the presence of MPs in every kind of environmental samples. Since 2014, the number of publications on MP contamination has increased rapidly (He et al. 2018; Borja and Elliott 2019), and the list is daily updated. The pathways that different MP types follow from sources to sinks and the dynamics of their transfer through the food web, as well as the complex patterns of biological effects on living organisms are the main knowledge gaps to be filled (Horton et al. 2017; Worm et al. 2017). However, the lack of standard analytical approaches and shared data reporting generates a lot of inconsistencies, reducing the information value and the comparability among different studies (Cowger et al. 2020a).

Most applied procedures to extract MPs from complex matrices — such as soils, sediments, or biological samples — imply a digestion step to eliminate the biogenic component (Stock et al. 2019). Thereafter, samples are usually filtered onto a membrane, where MPs are identified using a stereomicroscope and their polymeric composition characterized through Fourier transform infrared spectroscopy (FT-IR) or Raman spectroscopy (Lenz et al. 2015). Finally, the collected MPs are almost always classified according to shape categories and size classes (Shim et al. 2017). The qualitative operator-based classification of MPs often implies loss of objective information. Visual identification is a laborious and time-consuming task, and therefore subject to observer bias (Primpke et al. 2017). Furthermore, MP categories used in different studies are not always congruent and clearly defined, resulting in the lack of a standard, globally shared glossary (Miller et al. 2021). Then, the use of categorical variables to describe the shape and size of MPs limits cross-studies consistency, and therefore the understanding of pathways, mechanisms, and patterns that describe the fate of different MP types within ecosystems (Cowger et al. 2020b).

The present study investigates the potential of two open-source image processing software in providing an accurate characterization of the shape of MPs using a restricted set of shape descriptors. The image processing software used are the shapeR package for R (Libungan and Pálsson 2015a) and ImageJ 1.52v (https://imagej.nih.gov/ij/, accessed 28 March 2022). To ascertain that the selected tools can measure small differences in the shape and size of MPs, we performed an experiment to verify the detection of shape variations in MPs treated with mildly corrosive chemicals. Since different chemicals attack different polymeric structures (Cole et al. 2014; Rocha-Santos and Duarte 2015), we treated six plastic polymers with different digestion protocols known to be slightly corrosive against MPs with various compositions. The selected polymers are the most common polymers found in environmental samples (nylon, NY; polyethylene, PE; polyethylene terephthalate, PET; polypropylene, PP; polystyrene, PS; polyvinylchloride, PVC) (Hidalgo-Ruz et al. 2012). The digestion protocols were chosen among the variety of methodological approaches used in the extraction of MPs from biological matrices.

The rationale for the experiment supposed that if the selected tools are useful to detect small modifications in MPs induced by the known corrosiveness of digestive solutions, the same tools could be used to obtain a careful characterization of the shape and size of MPs based on shape descriptors. The development of new tools for translating categorical data into quantitative variables can improve current methods for the characterization of the shape and size of MPs, providing a rigorous methodological framework for monitoring routines that will be essential for effective management policies (Hardesty and Wilcox 2017; Valente et al. 2020). MPs are usually classified according to shape categories that at times provide information on their origin (Rochman et al. 2019; Miller et al. 2021). However, different studies often adopt different categorization systems due to the lack of standard definitions (Hartmann et al. 2019; Yu et al. 2022). In this view, shape descriptors could be the base of a consistent glossary for microplastic classification, which will be crucial to understand the relative importance of different MP sources and thus to guide appropriate mitigation actions (Rochman et al. 2016). Furthermore, image analysis might help the validation of new procedures for the extraction of microplastics from complex matrices.

## Materials and methods

Three digestion protocols, which share a short incubation time (about 12 h), were selected according to the use of different digestive solutions at different incubation temperatures, namely: 10% KOH at 60 °C (hereafter KOH; Rochman et al. 2015); 30% H_2_O_2_ at 50 °C (H_2_O_2_; Li et al. 2016, modified according to Bianchi et al. 2020); and 15% H_2_O_2_ + 5% HNO_3_ at 40 °C (HNO_3_; Bianchi et al. 2020). A treatment with Milli-Q ultrapure water at room temperature (CTRL; H_2_O at 25 °C) was set as control treatment.

KOH was produced by dissolving KOH pellets (Carlo Erba Reagents) in ultrapure Milli-Q water. H_2_O_2_ was purchased from Carlo Erba Reagents (Italy). HNO_3_ solution was prepared by diluting 30% H_2_O_2_ and 65% HNO_3_ (AnalaR NORMAPUR® analytical reagent, VWR chemicals) with ultrapure Milli-Q water.

Following Nuelle et al. (2014) and Bianchi et al. (2020), all MPs were produced by fragmenting daily-use plastic products or laboratory materials (NY: black plastic cable ties; PE: blue vials cap; PET: light blue water bottle; PP: black pen cap; PS: red plastic cup; PVC: orange pipe) using a variety of tools (such as scissors, pincers, clippers, and graters) to obtain different breaking profiles. The polymer composition of all materials was verified using a Nicolet iS10 Fourier Transform Infrared Spectroscopy with attenuated total reflection (ATR) FT-IR (Thermo Fisher Scientific, Madison, WI, USA). The plastic products selected for analysis had spectra matching at 81–97% with spectra of reference libraries (“HR Spectra Polymers and Plasticizers by ATR”, and “HR Polymer Additives and Plasticizers”) provided with OMNIC 9.8.286 (Thermo Fisher Scientific Inc.).

**Fig. 1:**
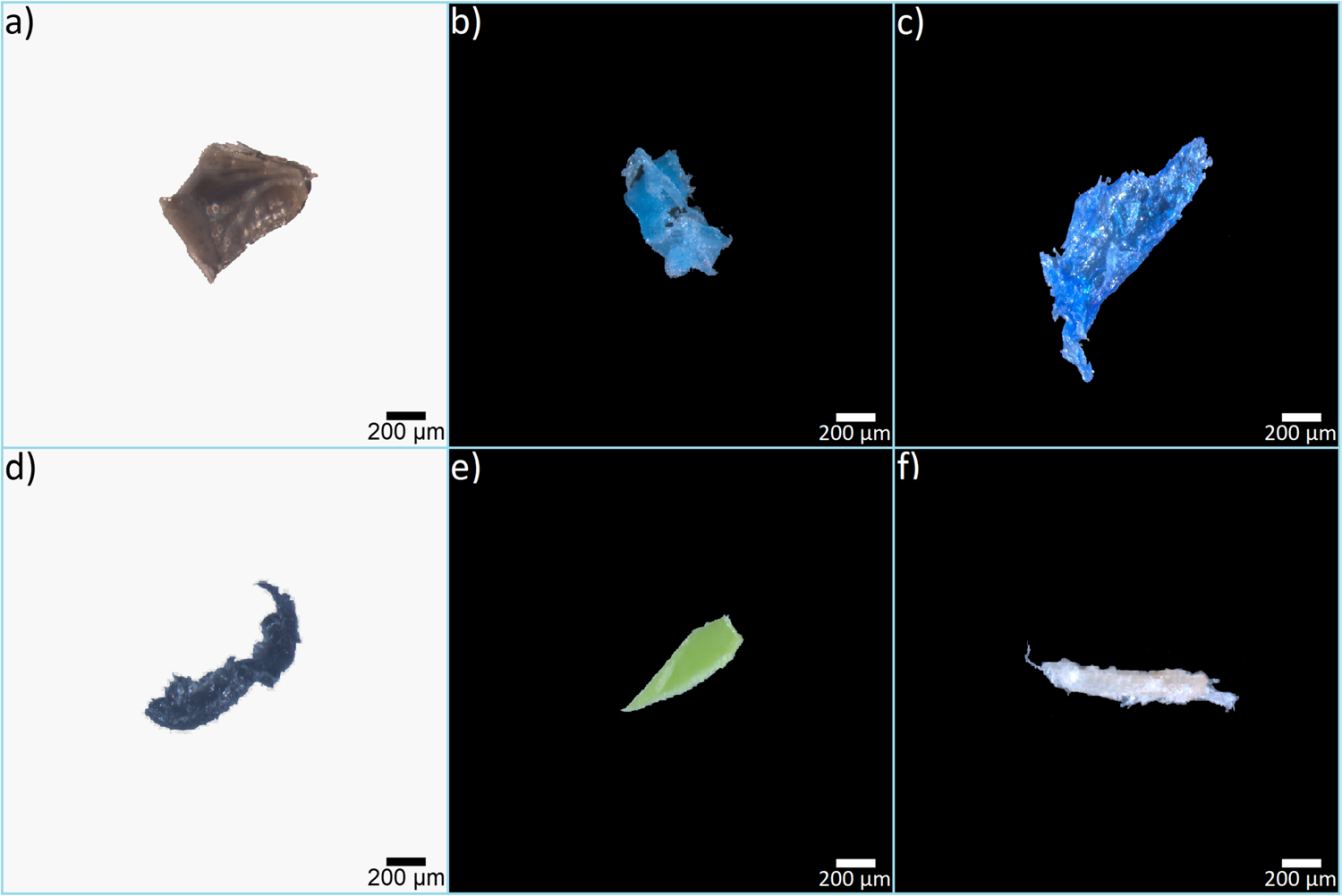
Materials. Representative images of microplastics produced by fragmenting daily-use plastic products or laboratory materials: (**a**) nylon (NY) from a black plastic cable tie; (**b**) polyethylene (PE) from a blue vial cap; (**c**) polyethylene terephthalate (PET) from a light-blue water bottle; (**d**) polypropylene (PP) from a black pen cap; (**e**) polystyrene (PS) from a green plastic cup; (**f**) polyvinylchloride (PVC) from an orange pipe. The polymer composition of all materials was verified using a Nicolet iS10 Fourier Transform Infrared Spectroscopy with attenuated total reflection (ATR) FT-IR (Thermo Fisher Scientific, Madison, WI, USA)

MPs for the analyses were chosen using a graphical filtering to select particles with surface area < 2 mm^2^ (size range: 0.171–1.874 mm^2^; representative images are available in Fig. 1). A total of 720 MPs (30 MPs ∙ 6 polymers ∙ 4 treatments) were photographed before and after the treatment using a camera-equipped stereomicroscope (see “Image capture and pre-processing”). Groups of 180 MPs (30 MPs for each polymer) were randomly assigned to the digestion and control treatments. Samples of 30 MPs were plunged into 2 ml of the abovementioned four solutions (i.e., three test and one control) and stored in a water bath at the established temperatures. At the end of the incubation time, MPs were recovered onto glass microfiber membranes (Whatmann GF/D™; 2.7 μm pore size) by filtering the digestive solutions using a vacuum pump system. Then, wet membranes were placed into individual glass Petri dishes and dried.

### Image capture and pre-processing

Pictures of MPs were taken before and after their treatment using a ZEISS Discovery.V20 SteREO modular stereo microscope with motorized zoom (objective PlanAPO S 1.0 x, FWD 60 mm; eyepieces WPL 10x/23 Br. foc, magnification 7.5 × –150 × , object field 30.7–1.5 mm), equipped with SYCOP 3 system control panel, EMS 3 controller, and an AxioCam ERc5s camera.

All the pictures were captured setting the zoom to 50 × , with the aid of motorized focusing and substantially constant background and lighting conditions for each polymer.

**Fig. 2:**
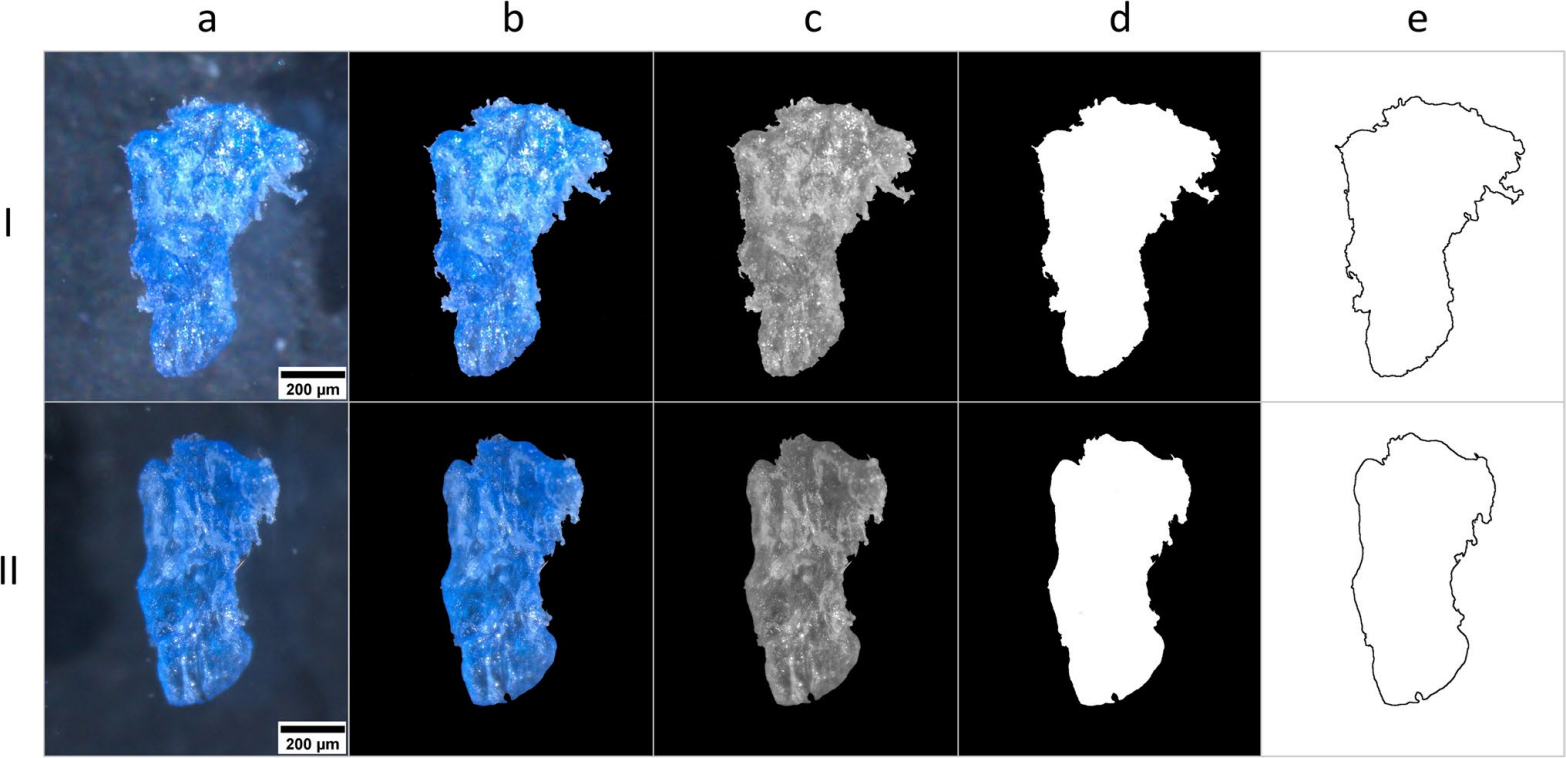
Image pre-processing. Representative pictures of the image elaboration process. Sample images: PET particle before (I) and after (II) a treatment with 10% KOH at 60 °C (incubation time ≈ 12 h). (**a**) Original images stored in full color; (**b**) pre-processing for reducing background noise and enhance the contrast (performed using Adobe Photoshop® version 19.1.6); (**c**) conversion to 8-bit images using ImageJ 1.52v (https://imagej.nih.gov.ij//, accessed 28 March 2022); (**d**) threshold selection for extracting the particle from the background; (**e**) outline detection

The 1440 obtained images (2560 ∙ 1920 pixel, 1143 pixel ∙ mm^−1^) were stored in full color in jpeg format. Following Libungan and Pálsson (2015b), an image manipulation program (i.e., Adobe Photoshop® version 19.1.6) was used to reduce background noise and enhance the contrast to simplify the outline detection with shapeR and the threshold selection for particle analysis with ImageJ (Cowger et al. 2020b). The image elaboration process is summarized in Fig. 2. Further details on the process, including scripts and processing time estimates, are available in Supplementary Information.

### Image processing

The shapeR package for R was created as a tool to analyze otolith shape variations among fish population, but it could be useful in studies of any two-dimensional objects. It was used to automatically extract the contour outline of each microplastic (see Script 1 in Supplementary Information). The considered outputs were surface area of each microplastic and their 64 wavelet coefficients (computed on 10 wavelet levels using the Daubechies least-asymmetric wavelet) (Gençay et al. 2001; Libungan and Pálson 2015b).

ImageJ is a widely used open-source program for scientific image processing. The automated use with Macros and Batch Processing was employed to get shape descriptors from each image (see Script 2 in Supplementary Information). The computed shape descriptors were: surface area; compactness (ratio of the particle’s area to the area of a circle with the same perimeter, 4π ∙ area ∙ perimeter^−2^); solidity (ratio of the area of an object to the area of the convex hull of the object, area ∙ convex area^−1^); and convexity (ratio of the perimeter of an object’s convex hull to the perimeter of the object itself, convex perimeter ∙ perimeter^−1^).

### Statistical analysis

To ensure the absence of strong corrosive effects, the recovery efficiency of each treatment was evaluated in terms of recovery rate (no. of treated MPs ∙ no. of recovered MPs^−1^) and pre-post pairing rate (no. of treated MPs ∙ no. of pre-post paired MPs^−1^). Surface area measurements obtained with shapeR and ImageJ were compared by fitting a linear regression model. Wavelet coefficients were used to graphically assess pre-post variation of the mean shape of each sample. Moreover, pre-post differences in Wavelet coefficients were analyzed using an ANOVA-like permutation test for Constrained Analysis of Principal Coordinates.

Then, effect size estimates (Glass’s delta, *Δ*) were used to assess pre-post variations in surface area, compactness, solidity, and convexity. Depending on data distribution (Shapiro–Wilk normality test) and homogeneity of variances (Levene test), either analysis of variance (ANOVA), or Kruskal–Wallis tests were performed to test the differences among the effects of treatments applied to each polymer (*α* = 0.05). When significant differences were detected, post-hoc comparisons (respectively Tukey’s HSD test, or Mann–Whitney *U* test with Bonferroni correction) were used to highlight the formation of treatment groups.

All statistical analyses were performed using R 4.0.3 (R Core Team 2020) and the packages shapeR (Libungan and Pálsson 2015a), lawstat (Gastwirth et al. 2020), agricolae (de Mendiburu 2020), and vegan (Oksanen et al. 2020). Graphical outputs were produced using Cairo (Urbanek and Horner 2020).

## Results

**Fig. 3:**
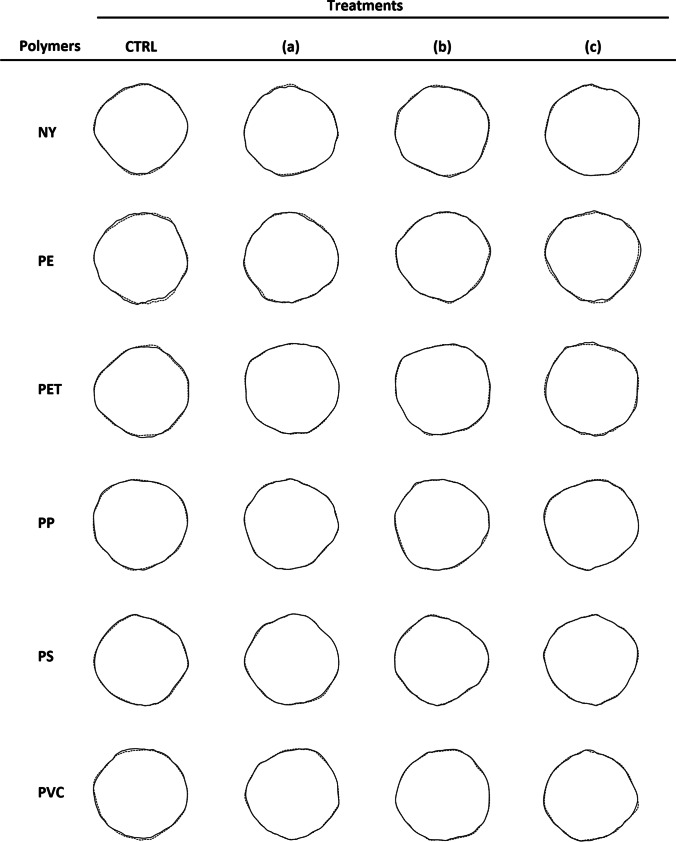
Mean shape differences. Graphical assessment of the mean shape variation of microplastics treated with three different digestion protocols (incubation time ≈ 12 h). A treatment with Milli-Q ultrapure water at room temperature (25 °C) was set as control treatment (**CTRL**). Tested polymers: nylon (**NY**), polyethylene (**PE**), polyethylene terephthalate (**PET**), polypropylene (**PP**), polystyrene (**PS**), and polyvinylchloride (**PVC**). Applied treatments: (**a**) 30% H_2_O_2_ at 50 °C, (**b**) 5% HNO_3_ + 15% H_2_O_2_ at 40 °C, (**c**) 10% KOH at 60 °C. Drawings are based on wavelet reconstruction: the solid lines represent the mean shape of microplastics before the treatment; the dashed lines highlight the deviations detected after the treatment

A 100% recovery rate was achieved for all the treatments. The pre-post pairing of all MPs was also completed. Surface area measurements obtained using shapeR and ImageJ were nearly perfectly fitted (*R*^2^ > 0.99). Both ANOVA-like permutations tests and the graphical assessment of mean shapes computed for each sample before and after the treatment showed the absence of significant shape variations (all *p*-values > 0.05; Fig. 3).

**Fig. 4:**
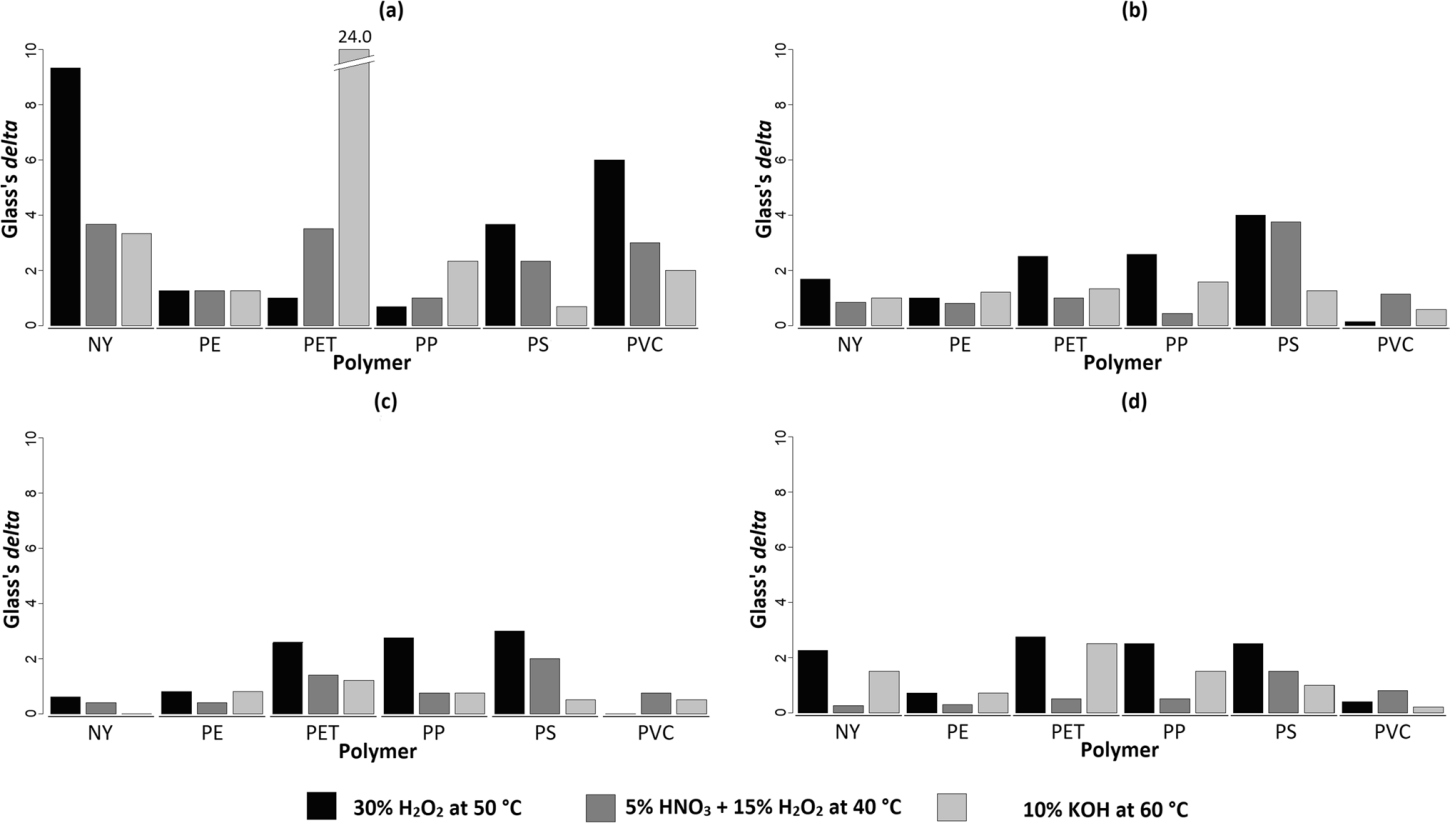
Effect size estimates. Bar plots representing Glass’s delta values computed for microplastics treated with three different digestion protocols (incubation time ≈ 12 h). A treatment with Milli-Q ultrapure water at room temperature (25 °C) was set as control treatment for effect sizes computing. Tested polymers: nylon (NY), polyethylene (PE), polyethylene terephthalate (PET), polypropylene (PP), polystyrene (PS), and polyvinylchloride (PVC). Applied treatments: 30% H_2_O_2_ at 50 °C (black bars), 5% HNO_3_ + 15% H_2_O_2_ at 40 °C (dark gray bars), 10% KOH at 60 °C (light gray bars). Shape descriptors: (**a**) surface area [mm^2^]; (**b**) compactness (ratio of the particle’s area to the area of a circle with the same perimeter, 4π ∙ area ∙ perimeter^−2^); (**c**) solidity (ratio of the area of an object to the area of convex hull of the object, area ∙ convex area^−1^); (**d**) convexity (ratio of the perimeter of an object’s convex hull to the perimeter of the object itself, convex perimeter ∙ perimeter.^−1^)

Despite the lack of strong shape differences, statistical testing in Table 1 highlighted significant surface area variations for: (1) PET particles treated with KOH; (2) NY, PS, and PVC particles treated with H_2_O_2_ (Table 1, a). The largest decrease in surface area was recorded for KOH-treated PET particles (0.061 ± 0.003 mm^2^; *Δ* = 24.0). NY resulted in the most susceptible polymer to the H_2_O_2_ treatment (*Δ* = 9.3), followed by PVC (*Δ* = 6.0), and PS (*Δ* = 3.7). PE (max *Δ* = 1.3) and PP (max *Δ* = 2.3) proved to be the most resistant polymers to the used treatments (Fig. 4).

**Table 1:** Shape variations. Differences in surface area, compactness, solidity, and convexity detected in microplastics treated with three different digestion protocols (incubation time ≈ 12 h). A treatment with Milli-Q ultrapure water at room temperature (25 °C) was set as control treatment (CTRL). Tested polymers: nylon (NY), polyethylene (PE), polyethylene terephthalate (PET), polypropylene (PP), polystyrene (PS), and polyvinylchloride (PVC). Shape descriptors: (**a**) surface area [mm^2^]; (**b**) compactness (ratio of the particle’s area to the area of a circle with the same perimeter, 4π ∙ area ∙ perimeter^−2^); (**c**) solidity (ratio of the area of an object to the area of convex hull of the object, area ∙ convex area^−1^); (**d**) convexity (ratio of the perimeter of an object’s convex hull to the perimeter of the object itself, convex perimeter ∙ perimeter^−1^). ANOVA (1) or Kruskal–Wallis test (2) was performed to test the differences among the effects of treatments applied to each polymer. When significant differences were detected, Tukey’s HSD test (1) or Mann–Whitney *U* test with Bonferroni correction (2) was used to highlight the formation of treatment groups. Values with the same superscript letter are not significantly different (*α* = 0.05)

Polymer	Treatment			
	CTRL	30% H_2_O_2_ at 50 °C	5% HNO_3_ + 15% H_2_O_2_ at 40 °C	10% KOH at 60 °C
*a) Surface area variations [mm*^*2*^*]* (1)				
NY	− 0.010 ± 0.003 ^b^	0.018 ± 0.003 ^a^	0.001 ± 0.004 ^b^	0.000 ± 0.003 ^b^
PE	0.008 ± 0.004	0.003 ± 0.003	0.003 ± 0.004	0.013 ± 0.003
PET	0.013 ± 0.002 ^b^	0.015 ± 0.002 ^b^	0.020 ± 0.002 ^b^	0.061 ± 0.003 ^a^
PP	0.003 ± 0.003	0.005 ± 0.002	0.006 ± 0.002	0.010 ± 0.002
PS	0.012 ± 0.003 ^b^	0.023 ± 0.002 ^a^	0.019 ± 0.003 ^ab^	0.014 ± 0.003 ^ab^
PVC	0.005 ± 0.002 ^b^	0.017 ± 0.002 ^a^	0.011 ± 0.002 ^ab^	0.009 ± 0.002 ^b^
*b) Compactenss variations* (2)				
NY	− 0.005 ± 0.006	0.005 ± 0.008	− 0.010 ± 0.007	− 0.011 ± 0.004
PE	− 0.004 ± 0.010	− 0.014 ± 0.006	− 0.012 ± 0.010	− 0.016 ± 0.010
PET	− 0.015 ± 0.006 ^ab^	0.000 ± 0.004 ^a^	− 0.009 ± 0.005 ^ab^	− 0.023 ± 0.008 ^b^
PP	− 0.002 ± 0.007	− 0.020 ± 0.005	− 0.005 ± 0.004	− 0.013 ± 0.004
PS	− 0.008 ± 0.004	− 0.024 ± 0.009	− 0.023 ± 0.006	− 0.003 ± 0.006
PVC	− 0.013 ± 0.007	− 0.012 ± 0.007	− 0.005 ± 0.005	− 0.009 ± 0.011
*c) Solidity variations* (2)				
NY	0.000 ± 0.005	− 0.003 ± 0.006	− 0.002 ± 0.004	0.000 ± 0.003
PE	− 0.003 ± 0.005	− 0.007 ± 0.003	− 0.005 ± 0.005	0.001 ± 0.004
PET	− 0.012 ± 0.005 ^ab^	0.001 ± 0.003 ^a^	− 0.005 ± 0.003 ^ab^	− 0.018 ± 0.005 ^b^
PP	0.002 ± 0.004	− 0.009 ± 0.004	− 0.001 ± 0.002	− 0.001 ± 0.002
PS	− 0.004 ± 0.004 ^ab^	− 0.016 ± 0.004 ^b^	− 0.012 ± 0.002 ^ab^	− 0.002 ± 0.002 ^a^
PVC	− 0.005 ± 0.004	− 0.005 ± 0.003	− 0.002 ± 0.002	− 0.007 ± 0.008
*d) Convexity variations* (2)				
NY	− 0.005 ± 0.004	0.004 ± 0.005	− 0.006 ± 0.004	− 0.011 ± 0.003
PE	− 0.004 ± 0.007	− 0.009 ± 0.005	− 0.006 ± 0.008	− 0.009 ± 0.005
PET	− 0.010 ± 0.004 ^ab^	0.001 ± 0.003 ^a^	− 0.008 ± 0.003 ^a^	− 0.020 ± 0.006 ^b^
PP	0.000 ± 0.004	− 0.010 ± 0.003	− 0.002 ± 0.003	− 0.006 ± 0.003
PS	− 0.005 ± 0.004	− 0.015 ± 0.006	− 0.011 ± 0.004	− 0.001 ± 0.005
PVC	− 0.007 ± 0.005	− 0.009 ± 0.005	− 0.003 ± 0.003	− 0.006 ± 0.007

Considering the other shape descriptors (i.e., compactness, solidity, and convexity), no significant differences were detected between CTRLs and the other treatments (Table 1, b–d). However, though the shape variations induced by the treatments were not extended (Δ mean ± sd = 1.27 ± 0.96), our results revealed slight modifications toward a more rounded shape (increases in compactness, solidity, and convexity values) of KOH-treated PET particles (as appreciable in the sample image in Fig. 2) and H_2_O_2_-treated PS particles. In particular, significant differences were noticed in: (1) compactness and solidity variations between PET particles treated with KOH and H_2_O_2_; (2) convexity variations between PET treated with KOH and the two oxidant solutions (i.e., H_2_O_2_ and HNO_3_); (3) solidity variations between PS particles treated with H_2_O_2_ and KOH. Overall, Glass’s delta values indicated H_2_O_2_ as the most impacting treatment on compactness, solidity, and convexity of MPs (ΣΔ_H2O2_ = 32.7, ΣΔ_HNO3_ = 17.5, ΣΔ_KOH_ = 18.1; Fig. 4b–d).

## Discussion

### Experimental results

In most studies, MPs are visually identified using a stereomicroscope and later classified by observers according to customary shape categories and size classes (He et al. 2018). Being manual classification a step that often implies loss of information, we explored the possibility to preserve the informative value by processing images caught by a camera-equipped stereomicroscope. To verify the usefulness of image processing, we exploited information from previous studies on the corrosive effect of various digestion protocols on different plastic polymers (Nuelle et al. 2014; Cole et al. 2014; Rocha-Santos and Duarte 2015; Dehaut et al. 2016; Karami et al. 2017; Bianchi et al. 2020). In this view, we verified the reliability of two different approaches for image processing (i.e., shapeR and ImageJ) by testing their power in detecting small shape variations in MPs treated with mildly corrosive digestion protocols.

The analysis of surface area variations returned results about the vulnerability of plastic polymers to digestive solutions that were aligned with most of the acquired knowledge. Image analysis highlighted no corrosive effects of HNO_3_, as reported by Bianchi et al. (2020). On the contrary, H_2_O_2_ seemed the most impacting treatment. Previous studies reported that H_2_O_2_ damages different plastic polymers. Karami et al. (2017) described non-optimal recovery rates for NY, PS, and PVC microplastics treated with 30% H_2_O_2_ at 50 °C for 96 h, highlighting possible changes of the polymeric structures of NY (decreased intensity of the peak at 1435 cm^−1^ and increased intensity of the band at 1118 cm^−1^), PS (sharpness of the peak at around 998 cm^−1^), and PVC (decreased intensity of the band for the stretching of C–Cl) through Raman spectroscopy.

Shape variations in PET particles treated with KOH were documented by Dehaut et al. (2016). In this case, other studies suggested that degradation may be led by the high incubation temperature of 60 °C, which approaches the softening point of PET (74–85 °C) (Wan et al. 2001). In fact, Karami et al. (2017) also described for temperatures ≥ 50 °C: (1) a reduction of KOH-treated PET recovery rates; (2) a greater number of voids on the surface of the particles (detected with scanning electron microscopy); (3) a decreased sharpness of the band at 1610 cm^−1^ (ring C = C stretching) (Awasthi et al. 2010). Overall, though the extent of the variations induced by the used treatments was not sufficient for determining strong shape differences in the MPs we examined, our results suggested that comparability among different studies could be affected by the different digestion protocols adopted during the extraction of MPs (Table 1, b–d), with a potentially more significant effect on MPs with smaller sizes.

### Methodological perspectives

Image processing seems a reliable tool for detecting small differences in the shape and size of MPs. Therefore, the characterization of MPs through image processing could be useful for many applications.

Firstly, as no standardized protocols exist to extract MPs (Miller et al. 2021), no standardized validation procedure of these protocols is also in use (for instance compare Nuelle et al. 2014; Avio et al. 2015; Karami et al. 2017; Bianchi et al. 2020). Since the efficiency of different digestion protocols changes according to the chemical composition of different environmental matrices (Bianchi et al. 2020), possibly new protocols will be developed in the future. Therefore, image processing could represent a replicable key step useful in detecting and quantifying the corrosiveness of digestive solutions on MPs with different sizes and polymer compositions.

Furthermore, image processing could drive the development of a standard glossary for MP classification. Visual identification is a basic approach for the quantification of MPs (He et al. 2018). However, it is inevitably affected by the staff training level and weariness caused by the labor-intensive and time-consuming activity (Cowger et al. 2020b). The computation of shape descriptors can bring to the development of a new and consistent MP classification system based on strict quantitative definitions, avoiding the influence of the observer bias. The main shape categories used to classify MPs worldwide are fiber, film, fragment, and sphere/pellet (Lusher et al. 2017). All these forms can be distinguished according to the combination of different shape descriptors. For instance, fibers are thin particles that are certainly characterized by high values of aspect ratio (height-to-width ratio, major axis ∙ minor axis^−1^; Cole 2016), elongation indexes (e.g., the width-to-length ratio of the object bounding box, bounding-box width ∙ bounding-box length^−1^; Wirth 2004; Primpke et al. 2019), and perimeter-to-surface area ratio. Diagnostic descriptors of spheres/pellets can be compactness (see “Image processing”) and roundness (inverted aspect ratio, minor axis ∙ major axis^−1^), while fragments can be distinguished from films by the presence of more crooked edges (GESAMP, 2019), and therefore by lower convexity values (see “Image processing”).

These and other numerical descriptors of shape may also allow the definition of additional categories and subcategories that could be established to answer specific research questions (GESAMP, 2019; Miller et al. 2021). For instance, Avio et al. (2020) pointed out that it is important to discriminate lines and filaments from textile microfibers to correctly evaluate the relative importance of different MP sources in marine areas exploited by fisheries. Lines and filaments derived from fisheries are defined as rod-like particles with regular diameter, while microfibers are characterized by a ribbon-like shape, not regular diameter, and frayed ends. Therefore, rectangularity measures (e.g., ratio of the surface area of the object to the surface area of the minimum bounding rectangle, surface area ∙ bounding box surface area^−1^; Rosin 1999) and curl indexes (length ∙ fiber length^−1^; Wirth 2004) can be used in distinguishing MP sub-types within the class of threadlike particles. The description of the shape and size of MPs using quantitative descriptors such as major and minor axes, surface area, perimeter, convex hull, and bounding box of each item could be a reliable way to ensure comparability among current studies, as well as a mode of preserving data for future analytical advances.

### Future advances

The potential of high-technology techniques used in the study of MP pollution is increasing through integration with image processing and analysis. Recent studies propose algorithms to automate MP detection and recognition using chemical imaging based on FT-IR or Raman microspectroscopy (Primpke et al. 2018; Anger et al. 2018). Recently, Serranti et al. (2018) successfully explored the utility of HyperSpectral Imaging in the characterization of ocean-floating MPs, while Chen et al. (2021) assessed the degradation degree of MPs through a spectral-image fusion model. Other authors already remarked that in-depth analyses of surface morphology can be useful to estimate the degradation degree of MPs (Veerasingam et al. 2016; Cai et al. 2018). Images can detect the presence and frequency of holes, hollows, and rifts. Therefore, roughness measures can be used to estimate the extent of the weathering process, providing interesting information on the persistence time of MPs in different environments (Cowger et al. 2020b).

Despite that all these new methodological approaches will contribute decisively to the comprehension of the environmental fate of MPs, research has the assignment of finding also low-cost systems that are suitable for environmental monitoring routines. From this perspective, holographic imaging coupled with machine learning is a promising approach (Bianco et al. 2020). Our study would contribute to this line of interest indicating that a lot of very informative data can be collected through widely accessible labware. The potential of image analysis from optical microscopy is currently underexploited in this field (Cowger et al. 2020b), but the development of new reliable approaches to describe MP pollution through image processing is a very hard task. The aim is to fairly well describe the shape of MPs using a restricted set of numbers. Although the shape cannot be redrawn from shape descriptors, these should be sufficiently different to distinguish different shapes. The quality of quantitative information obtained from images strictly depends on the quality of the original image and the goodness of pre-processing (Wirth 2004). Therefore, further studies focusing on the definition of general measurement rules will be very important (Cowger et al. 2020b). Although fibers, filaments, and films naturally exhibit their two largest dimensions when placed on a plain, this may not be true for MP fragments with non-negligible thickness and irregular margins. Ensuring the repeatability of 2D image-based measurements may be difficult for this type of MPs. Standardizing measurement conditions (such as considering only maximum sizes even for particles with a complex 3D structure) can represent a simple way to reduce this source of bias. However, novel approaches based on extended depth of field (EDF) processing and other thickness estimation methods will need to be explored to find more reliable ways to describe the 3D aspect of MPs. Moreover, further efforts should be addressed in developing tools to consistently describe even colors, opacity, and texture of MPs (Maes et al. 2017; Rochman et al. 2019).

## Conclusions

Microplastics are hazardous and atypical contaminants that may differ in size and shape. Current methods to characterize the shape of microplastics are based on visual identification, which is inevitably affected by observer bias. Moreover, the lack of a globally shared glossary for the classification of microplastics often implies the loss of comparability among different studies. We tested and discussed the potential of image processing in providing new tools for describing the shape and size of microplastics through quantitative shape descriptors. Our results suggest that image analysis can allow an accurate characterization of the shape of microplastics by using widely accessible labware and open-source software. Novel analytical methods exempt from subjective bias will contribute decisively to the development of consistent guidelines for studies on environmental contamination by microplastics. In this view, image processing is a branch of computing and information science that will have to be more included among the variety of disciplines involved in the study of microplastic pollution.

## Supplementary Information

Below is the link to the electronic supplementary material.

Supplementary file1 (PDF 486 KB) Further details on the image elaboration process, including illustrative scripts and processing time estimates, are available as Online Resource.

## Data Availability

The datasets generated during and/or analyzed during the current study are available from the corresponding author on reasonable request.
