# Supplementary material for: Image processing tools in the study of environmental contamination by microplastics: reliability and perspectives
Source: Environ Sci Pollut Res Int. 2022 Jul 28;30(1):298–309. doi: 10.1007/s11356-022-22128-3 (PMC9813107; doi:10.1007/s11356-022-22128-3)
Supplement: Supplementary file 1 — Supplementary file1 (PDF 486 KB) Further details on the image elaboration process, including illustrative scripts and processing time estimates, are available as Online Resource. [file 11356_2022_22128_MOESM1_ESM.pdf]

Image processing tools in the study of environmental contamination by microplastics: Reliability and perspectives

Tommaso Valente[1,2*], Daniele Ventura[1], Marco Matiddi[2], Alice Sbrana[2,3], Cecilia Silvestri[2], Raffaella Piermarini[2], Carlo Jacomini[2], Maria Letizia Costantini[1].

[1]'La Sapienza' University of Rome, Department of Environmental Biology, P.le Aldo Moro 5, 00185, Rome, RM, Italy.
[2]ISPRA, Italian National Institute for Environmental Protection and Research, Via di Castel Romano 100, 00128, Rome, RM, Italy.
[3]PhD Program in Evolutionary Biology and Ecology, Department of Biology, University of Rome 'Tor Vergata', Via della Ricerca Scientifica snc, 00133, Rome, RM, Italy.

***Corresponding author: Tommaso Valente**
University of Rome 'La Sapienza'
Department of Environmental Biology
5 P.le Aldo Moro
Rome, RM, ITALY 00185
E-mail: tommaso.valente@uniroma1.it
Tel.: +39 0649914773
Fax: +39 0649914773

Table of contents

Workflow diagram
Script 1 – Image Processing using shapeR
Phase I – Image archiving
Phase II – Data files preparation
Phase III – Image processing
Script 2 – Computation of shape descriptors using ImageJ
Processing times

Supplementary Information

This supplementary document provides a brief guide to the image elaboration process. The workflow diagram shows an overview of the process. The Script 1 section illustrates the use of the shapeR package (including image archiving rules and data files preparation). The Script 2 section describes the employment of macros in ImageJ. The last section provides an estimation of the processing times.

Workflow diagram

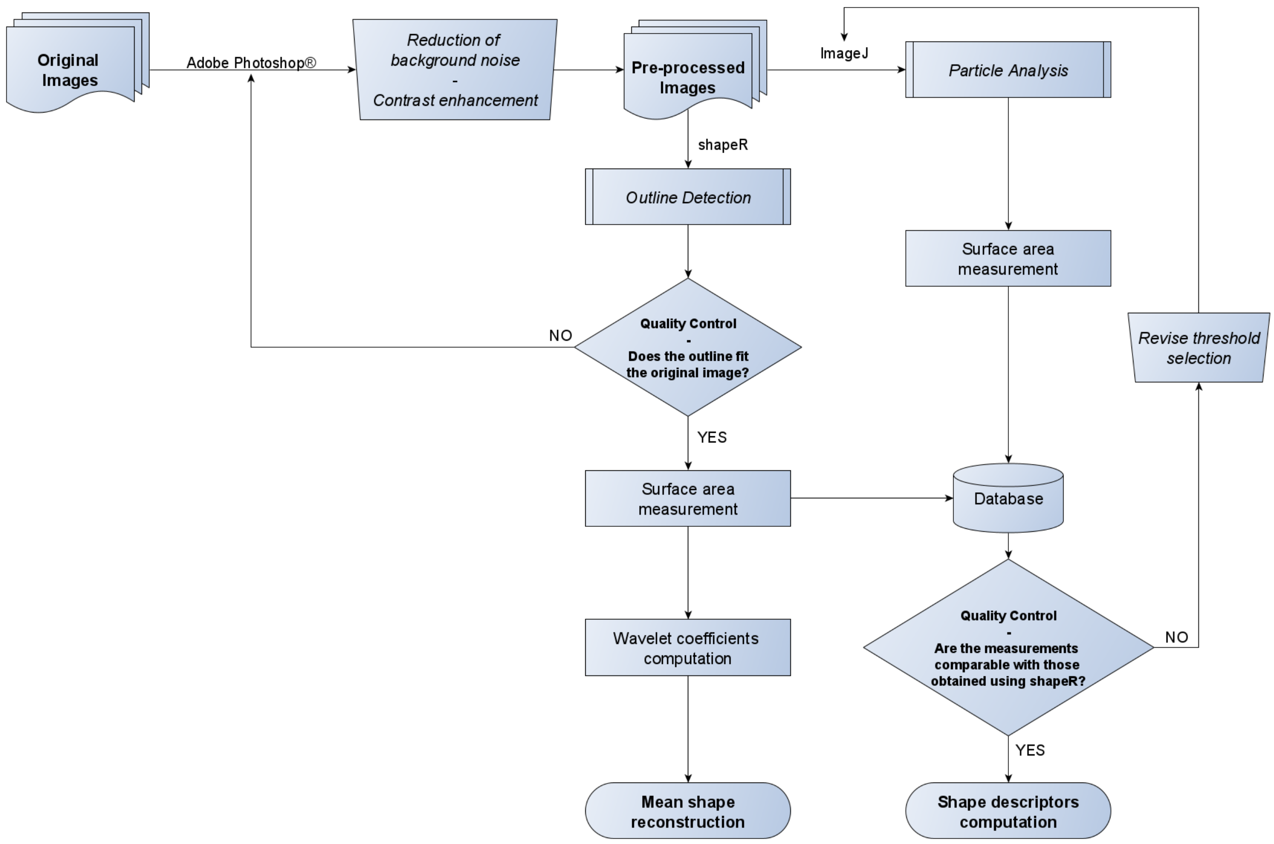

Script 1 – Image processing using shapeR

Phase I - Image archiving

The shapeR package requires images to be archived according to the following steps:

i) Images of each sampling unit must be grouped in a single folder. For instance, in our study we grouped all the images of KOH-treated nylon particles in a folder called "NYKOH_post", and the images of the same particles before their treatment in a folder called "NYKOH_pre".

ii) Store the sampling unit folders in a folder called "Original".

iii) Create a copy of "Original" called "Fixed".

iv) Include "Original" and "Fixed" in a folder called "ShapeAnalysis".

Phase II – Data files preparation

Data for each image must be provided in a *.csv file stored in "ShapeAnalysis". In this file, each row corresponds to an image and the following variables (columns) should be included:

- **pop**: name of the sampling unit (*e.g.*, pre or post);
- **folder**: folder name (*e.g.*, NYKOH_post or NYKOH_pre);
- **picname**: file name (*e.g.*, NYKOH_post_01, NYKOH_post_02, *etc.*);
- **cal**: calibration measurement for setting the scale (*e.g.*, pixel/mm).

The columns "folder" and "picname" are mandatory.

Phase III – Image processing

Following is reported an illustrative R script that allows the collection of surface area measurements in a *.csv file. The last command produces a mean shape reconstruction based on wavelet coefficients. It is sufficient specify the file paths at lines 4, 6 and 29, and then run the script in R after installing the shapeR package.

NB: *This script is optimized for the pictures analyzed in the present study (2560 · 1920 pixel, 1143 pixel · mm^{-1}). The default options require the particle to be placed in the center of the image. Adapting the script to images with different standards requires some modifications. Comments and suggestions in the code start with "#" and are highlighted in bold. For more information see Libungan and Pálsson (2015)* [https://doi.org/10.1371/journal.pone.0121102].

```
1  ##Request the package
2  library(shapeR)
3  #Select directory, specifying the info file name
4  example=shapeR("C:/[…]/ShapeAnalysis","infofile.csv")
5  #load the info file name
6  infofile=read.csv("C:/[…]/ShapeAnalysis/infofile.csv")
7  
8  #Remove residual noise
9  example=smoothout(example)
10 
11 #Perform outline extraction
12 #Default options for autothreshold are selected
13 #The argument "write.outline.w.org=FALSE" allows for faster analyses
14 #If "TRUE" it generates an "Original_with_outline" folder in "ShapeAnalysis"
15 #The images in "Original_with_outline" allows for quality control by showing the fit of the outline to the original image
16 example=detect.outline(example,write.outline.w.org=T)
17 
18 #Generate shape variables based on Wavelet coefficients
19 example=generateShapeCoefficients(example)
20 #Link the info file to the coefficients
21 example=enrich.master.list(example)
22 #Save surface area measurements in "results.csv" (used for comparison with the data from ImageJ)
23 area=data.frame(
24 cbind(
25 infofile$picname,
26 round(getMeasurements(example)[,1],3)))
27 names(results)=c("Sample","Area [mm^2]")
28 #Specify the path for saving the results
29 write.csv(results, file="C:/[...]/results.csv")
30 #Plot the mean shape reconstruction based on wavelet coefficients
31 #Make a comparison among "Original" subfolders
32 plotWaveletShape(example,"folder")
```

Script 2 – Computation of shape descriptors using ImageJ

Following is reported an illustrative script to obtain shape descriptors (*i.e.*, surface area, compactness, solidity, and convexity) from a set of images stored in the same folder and export the results in a *.csv file. The script was developed starting from the macro code "ConvexitySolidarity.txt" available at https://imagej.nih.gov/ij/macros, which requires ImageJ 1.31g. Since the particles were photographed on standard background and interferences were further reduced using an image manipulation program (*i.e.*, Adobe Photoshop® version 19.1.6; see Fig. 2 in the main text for a sample image), the extraction of the particles was performed by setting up an automatic threshold routine. A smoothing function was also applied to remove residual noise around the edges of the particles.

NB: *This script is optimized for the pictures analyzed in the present study (2560 · 1920 pixel, 1143 pixel · mm^{-1}). The default options require the particle to be placed in the center of the image. Adapting the script to images with different standards requires some modifications. Comments and suggestions in the code start with "//" and are highlighted in bold. For more information, codes, and tutorials visit https://imagej.nih.gov/ij*

```
1 // Specific default options for this script:
2 // 1) AutoThreshold require dark background
3 // 2) No background noise
4 // 3) Particle placed in the center of the image (2560 · 1920 pixel, 1143 pixel/mm)
5 // Results: Sample, Surface area [mm^2], compactness, solidity, and convexity
6 // Go to "Process > Batch > Macro…", select the input folder containing the images
7 // Paste the lines 9:13 in the text window and press "Process"
8 // Changes to line 10 allow for the definition of different thresholding routines
9 run("8-bit");
10 run("Smooth");
11 setAutoThreshold("Default dark");
12 run("Set Measurements...", "area perimeter shape display redirect=None decimal=3");
13 run("Analyze Particles...", "display");
14 // Go to "Plugins > New > Macro"
15 // Paste lines 18:53 in the new window
16 // After specifying the path for saving the results (line 53), go to "Macros > Run Macro" or press "CTRL+R"
17 // Results will be available at the end of the computation phase
18 n = nResults;
19 sample = newArray(n);
20 area = newArray(n);
21 perim = newArray(n);
22 circularity = newArray(n);
23 solidity = newArray(n);
24 for (i=0; i<n; i++) {
25 sample[i] = getResultLabel(i);
26 area[i] = getResult('Area', i);
27 perim[i] = getResult('Perim.', i);
28 circularity[i] = getResult('Circ.', i);
29 solidity[i] = getResult('Solidity', i);
30 }
31 run("Clear Results")
32 // Changes to lines 34:35 allow for the definition of different thresholding routines
33 // Changes to the command "doWand(x,y)" allow for the convex hull selection of not centered particles: select the xy coordinates of the object
34 run("Macro... ", "output_format=TIFF file=[] text1=[run(\"8-bit\");\nrun(\"Smooth\");\nsetAutoThreshold(\"Default dark\");\ndoWand(1280,
35 960);\nrun(\"Convex Hull\");\nrun(\"Measure\");\n\n]");
36 chperim = newArray(n);
37 for (i=0; i<n; i++) {
38 chperim[i] = getResult('Perim.', i);
39 }
40 run("Clear Results")
41 // Apply the appropriate scale definition at line 43 (e.g., pixel/mm)
42 // Omitting "scale" at lines 43 and 46 return results in pixel for a later image-by-image scaling
43 scale = 1143
44 for (i=0; i<n; i++) {
45 setResult("Sample", i, sample[i]);
46 setResult("Area [mm^2]", i, area[i]/(scale*scale));
47 setResult("Compactness", i, circularity[i]);
48 setResult("Solidity", i, solidity[i]);
49 setResult("Convexity", i, chperim[i]/perim[i]);
50 }
51 selectWindow("Results");
52 //specify the path for saving the results
53 saveAs("Results", "C:/[…]/results.csv");
```

Processing times

The time effort needed for image processing is strongly linked to the quality of the original image. The following table shows time estimates for image processing performed using a computer with operating system Windows 11 and an Intel Core i7-8565U CPU 1.8 GHz Processor with Turbo Boost up to 4.6 GHz. Since the particles analyzed in this study were photographed on standard background, the required time was rather reduced. To ensure quality control, all the pictures collected were analyzed by setting up the "Write image with outline" option on TRUE.

Image processing phase	Processing time ($s \cdot image^{-1}$)
Image manipulation (reduction of background noise)	~ 90
Computation of shape descriptors using ImageJ	0.93
Outline detection with shapeR	
- Write image with outline: TRUE	32.60
- Write image with outline: FALSE	1.00
